# Best period to replace or change plastic stents with self-expandable metallic stents using multivariate competing risk regression analysis

**DOI:** 10.1038/s41598-020-70081-3

**Published:** 2020-08-04

**Authors:** Masafumi Chiba, Masayuki Kato, Yuji Kinoshita, Nana Shimamoto, Youichi Tomita, Takahiro Abe, Yosuke Kawahara, Seita Koyama, Keisuke Kanazawa, Kazuki Takakura, Shintaro Tsukinaga, Masanori Nakano, Yuichi Torisu, Hirobumi Toyoizumi, Keiichi Ikeda, Hiroshi Arakawa, Kazuki Sumiyama

**Affiliations:** 1 Department of Endoscopy, The Jikei University School of Medicine, 3-25-8, Nishi-Shimbashi, Minato-ku, Tokyo, 105-8461 Japan; 2 Division of Gastroenterology and Hepatology, Department of Internal Medicine, The Jikei University School of Medicine, Tokyo, Japan

**Keywords:** Gastrointestinal diseases, Biliary tract disease, Pancreatic disease

## Abstract

In endoscopic biliary drainage (EBD) for various benign and malignant biliary disorders, the appropriate timing to replace or change a plastic stent (PS) with a self-expandable metallic stent (SEMS) remains unclear. This study aimed to define the best period to replace or change a PS with a SEMS. Between January 1, 2012, and December 31, 2018, 1,887 consecutive EBD procedures, including 170 SEMS placements, were retrospectively identified. The period to recurrent biliary obstruction (PRBO) was estimated and compared between the malignant and benign groups and according to each disease using time to event analysis and competing risk analysis. Compared with the benign group, the malignant group had significantly shorter median PRBO with interquartile range (IQR) after PS placement [108 (39 – 270) vs. 613 (191 – 1,329) days, P < 0.001], even on multivariate analysis, with a subdistribution hazard ratio (SHR) of 3.58 (P < 0.001). The shortest PRBO distribution from the first quartile of the non-RBO period was seen in Mirizzi syndrome cases (25 days, P = 0.030, SHR = 3.32) in the benign group and in cases of pancreatic cancer (32 days, P = 0.041, SHR = 2.06); perihilar bile duct cancer (27 days, P = 0.006, SHR = 2.69); and ampullary cancer (22 days, P = 0.001, SHR = 3.78) in the malignant group. Our study supports that stent replacement for the benign group is feasible after 6 months, and the best period to replace or change a PS with a SEMS should be decided on the basis of the underlying disease to prevent RBO.

## Introduction

Endoscopic biliary drainage (EBD) procedures are indispensable options in patients with acute cholangitis or an obstructive jaundice in both benign and malignant diseases^1–4^. In benign biliary disorders, temporary placement of a plastic stent (PS) has been useful for bile duct stones^5,6^, postoperative biliary leaks^5,7^, and benign biliary strictures with multiple PS placement^1,8^. In malignant biliary disorders, particularly in extrahepatic biliary strictures and inoperable perihilar strictures, self-expandable metallic stent (SEMS) has the advantage of a longer patency period, compared with PS^9–13^. However, PS is usually used in many cases because of the usability from the cost effectiveness or technical aspect^1,3,14–19^. Conversely, the development of recurrent biliary obstruction (RBO) after PS placement for malignant diseases could prevent chemotherapy or surgical therapy or worsen the patients’ quality of life or induce life-threatening complications associated with acute cholangitis^1,14,20–22^.

Up to the present time, the recommended period for PS replacement in malignant cases has been 3−6 months, based on studies that evaluated and compared the period to RBO (PRBO) between PS and SEMS^13,16^. Despite this recommendation, the actual period to recurrent biliary obstruction (PRBO) frequently seemed to be shorter in daily medical practice^23^. Likewise, the appropriate interval for PS replacement in benign diseases had been unclear. Because few studies on PS have reported details on the suitable interval or timing of replacement or change to SEMS according to the risk factors for RBO in each biliary disorder, the actual PRBO is not known in the field^1,9^. Therefore, the primary aim of this study was to define the best period to replace or change a PS with a SEMS in each disease, after resolving the risk factors for RBO.

## Materials and methods

### Study design

The present retrospective cohort study investigated PRBO after PS and SEMS placement. This study complied with “the TOKYO criteria 2014” for a time to event analysis with “the International consensus statements for endoscopic management of distal biliary stricture” and with the other guidelines on survival analyses^1,22,24,25^.

### Patients

Consecutive patients with suspected hepatobiliary–pancreatic disorders who underwent EBD attempts between January 1, 2012 and December 31, 2018 were retrospectively included in this study.

**Figure 1:**
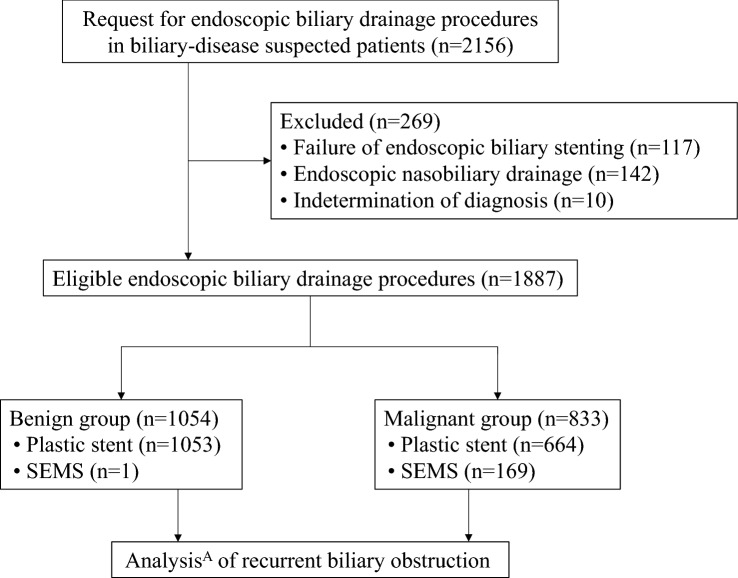
Study design. The patients eligible for inclusion in the study were those who underwent endoscopic biliary drainage with plastic stent or self-expandable metallic stent. ^A^Competing risk analysis and Kaplan–Meier method. *SEMS* self-expandable metallic stent.

Regarding eligibility, the population of stent placement included consecutive patients after PS placement. In preoperative stent placement, patients who received neoadjuvant chemotherapy were excluded from the accountment of the time to surgery. We excluded the cases of failure of endoscopic biliary stenting, endoscopic nasobiliary drainage, and indetermination of diagnosis (Fig. 1). The cases of duodenal stricture were also all excluded.

Data on endoscopic retrograde cholangiopancreatography (ERCP) procedures were retrieved from the Jikei University ERCP database, which was updated immediately after each procedure and contained data on > 6 months of follow up. All patients provided written informed consent to undergo EBD; they were aware of the opportunity to opt out of study participation (opt-out method of informed consent) because this study was an observational research. This study was approved by the Human Subjects Committee of Jikei University School of Medicine [ID no. 31-099 (9598)] and was subsequently registered with the University Hospital Medical Information Network Clinical Trials Registry (identification no. UMIN000037640). This study was conducted in accordance with the ethical principles of the Declaration of Helsinki (Fortaleza revision).

### Stent placement

PS used was of the 7-, 8.5-, or 10-Fr straight tube type (Flexima/Advanix J; Boston Scientific, USA and Japan or Quick Place V; Olympus Medical Systems, Tokyo, Japan). The length (5–15 cm) was chosen by the stricture location from the papilla. In some cases, a 7-Fr (5–12 cm) inside stent (CATHEX; Gadelius Medical, Tokyo, Japan or Advanix J; Boston Scientific) was needed.

In bile duct stone cases, PS without stone extraction was usually installed at first ERCP session in this study because patients with bile duct stone usually had on going acute cholangitis and/or obstructive jaundice. After the cholangitis or jaundice was cured, stent removal and stone extraction were performed at second ERCP session, which was considered right censoring^22^. Preoperative placement of PS was also included, and after surgeries, such as cholecystectomy, stent removal and stone extraction were performed at second ERCP session. The other cases included patients with severe complications who had difficulty undergoing cholecystectomy and stone extraction using EST and/or EPBD. In such situations, plastic stents were installed alone, and when RBO occurred, we exchanged the plastic stents.

If a spontaneous passage of bile duct stones was identified during ERCP, the patients with cholelithiasis underwent biliary stent placement to prevent the possible recurrence of choledocholithiasis owing to the gallstone passing through the common bile duct before cholecystectomy. As for reason, some patients with cholelithiasis underwent biliary stent placement.

When endoscopic drainage was required in Bismuth types II–IV strictures, the drainage of ≥ 50% of the liver volume was attempted^5^. If the bilateral drainage was difficult, unilateral drainage was attempted, prioritizing the bigger portion of liver volume.

### ERCP procedures

ERCP was performed under fluoroscopic view by experts who have performed > 200 ERCPs per year or by trainees with experts’ interference, depending on the situation. All patients who underwent ERCP were conscious but sedated with intravenous midazolam and pethidine administration during the ERCP procedure.

In almost all cases of stone extraction, endoscopic sphincterotomy (EST) with medium incision was performed. After stent placement in patients with bile duct stone, we did not prescribe ursodeoxycholic acid. In all cases of multiple PS placement, EST with small incision was performed. When medium incision of EST could not be performed owing to periampullary diverticulum or intradiverticular papilla, small incision of EST and balloon sphincteroplasty were performed. All procedures of stone extraction were conducted by the stone lithotripsy method. In cases of bile duct strictures, brush cytology and/or intraductal biopsies, in some cases using a spyglass, were performed during ERCP to exclude malignancy.

### Definitions of follow up and RBO factors

Based on “the TOKYO criteria 2014”, the recurrent biliary obstruction (RBO) was defined as a composite endpoint of either symptomatic occlusion or symptomatic migration, and the PRBO was defined as the time from SEMS/PS placement to the RBO^22^. The consecutive patients were followed up until June 30, 2019 by right censoring^25^. Patients were censored if they were lost to follow up without RBO, had asymptomatic migration on the day of routine replacement (i.e., non-cholangitis and/or non-jaundice), or when the stent was extracted during operation^22^. In this study, the independent variables of RBO risk factors were defined as age, sex, body mass index, antithrombotic agents, serum total bilirubin, grade of acute cholangitis, periampullary diverticulum, intradiverticular papilla, SEMS, the diameter and type of PS, total number of stentings, location and length of the biliary stricture, types of major papilla, altered gastrointestinal anatomy, and malignant and benign group. Time to surgery was defined as the duration from the initial day of ERCP to the day of the surgery. The diagnostic criteria and severity grading of acute cholangitis were based on the TOKYO Guidelines 2018^20^. The other definitions for PRBO, functional success, and severity grading of adverse events were based on the TOKYO criteria 2014 and International consensus statements for endoscopic management of distal biliary stricture^1,22^. Mirizzi syndrome was diagnosed by surgery, or when surgical resection was not indicated, ERCP and magnetic resonance cholangiopancreatography (MRCP) were used^26^. Bile duct stone clearance was verified by MRCP and blood test with no symptoms after stone extraction.

### Endpoints

The primary endpoint of the present study was PRBO in each disease. The secondary endpoints were (1) comparison of the RBO factors between the benign and malignant groups; (2) resolution of the risk factors for RBO in the benign and malignant groups; and (3) evaluation of the risk factors in each disease.

### Statistical analysis

PRBO was estimated using the Kaplan–Meier analysis and compared between the benign and malignant groups using the log-rank test. The event of patient death was treated as a censor. In the multivariate analysis, to avoid imbalances when there were seven or fewer events (= RBOs) per confounder, the number of dependent variables was adopted within the number of dependent variables / 7 items^27^. In the analysis, Fine and Gray model, which is based on a subdistribution hazard model (SHR)^28^, was used for competing risk regression analysis. To include death in the informative censoring for potential RBO in this model, death without RBO was treated as a competing risk. In this situation, Gray test was used for comparison of PRBO between the benign and malignant groups or within a group. After the initial multivariate analysis, the significant variables were adopted into the next multivariate analyses in each disease.

When appropriate, data were presented as mean (standard deviation: SD) or frequencies. The benign and malignant groups were compared using the Chi-square test or Fisher’s exact test for the proportions of categorical variables (e.g., technical success rates) and the Mann–Whitney U-test for the mean values of the continuous variables (e.g., time to 50% decrease or normalization of the bilirubin level). Missing values were excluded for complete case analysis. Two-sided P < 0.05 was considered significant. All analyses were performed using Stata version 15 (StataCorp LP; College Station, TX, USA).

## Results

### Patients

In the setting of a retrospective continuous series, 1,887 eligible patients who underwent EBD procedures using PSs or SEMSs were enrolled in the present study (Fig. 1 and Table 1). No significant differences in age and sex were noted between the benign and malignant groups. However, the proportion of cases with acute cholangitis at the initial EBD was significantly higher in the malignant group than in the benign group (54.7% vs. 48.8%, P = 0.04). In the malignant group, the mean time to surgery was 29.0 days (Table 1). PS placement after balloon sphincteroplasty only, large incision of EST, and multiple placement of PS without EST (small incision) were unintentionally not included in this series.

**Table 1:** Characteristics of endoscopic biliary drainage (n = 1887).

	Benign Group (n = 1,054)	Malignant Group (n = 833)	*p* value
Mean (range) age (years)	70.5 (21–99)	71.5 (21–96)	0.72^a^
No. men	683 (64.8)	532 (63.9)	0.67^b^
Body mass index, mean ± SD	22.4 ± 4.1	21.2 ± 4.2	< 0.001^a^
Antithrombotic agents	174 (16.5)	115 (13.8)	0.10^b^
Total bilirubin, mg/dL, mean ± SD	3.2 ± 4.0	5.7 ± 6.1	< 0.001^a^
**Acute cholangitis**^**c**^	391 (37.8)	525 (63.6)	< 0.001^b^
Grade I (Mild)	250 (24.2)	320 (38.7)	
Grade II (Moderate)	111 (10.7)	171 (20.7)	
Grade III (Severe)	30 (2.9)	34 (4.1)	
At initial EBD	241 (48.8)	180 (54.7)	0.041^b^
Periampullary diverticulum	285 (27.8)	102 (12.2)	< 0.001^b^
Intradiverticular papilla	42 (4.1)	8(1.0)	< 0.001^b^
**Type of major papilla**			< 0.001^b^
Naïve papilla	436 (41.4)	286 (34.3)	
Post EST^d^	92 (8.5)	72 (8.6)	
Post plastic stenting	518 (49.1)	427 (51.3)	
Other^e^	8 (0.8)	48 (5.8)	
**Location of biliary stricture**			< 0.001^f^
Distal	94 (8.9)	424 (50.9)	
Perihilar	70 (6.6)	339 (40.7)	
Intrahepatic	2 (0.2)	1 (0.1)	
Diffuse	4 (0.4)	3 (0.4)	
Non-stricture	884 (83.9)	66 (7.9)	
Length of stricture^g^, mm, Mean ± SD	24.1 ± 14.8	25.0 ± 13.5	< 0.001^a^
**Altered gastrointestinal anatomy**			< 0.001^f^
Normal	974 (92.4)	793 (95.2)	
Billroth I	56 (5.3)	15 (1.8)	
Billroth II	12 (1.1)	5 (0.6)	
Pancreaticoduodenectomy	0 (0)	9 (1.1)	
Roux-en-Y with gastrectomy	7 (0.7)	6 (0.7)	
Roux-en-Y with hepaticojejunostomy	2 (0.2)	0 (0)	
Other^h^	3 (0.3)	5 (0.6)	
**Diameter of plastic stent**^**i**^			< 0.001^b^
7 Fr	519 (49.2)	464 (55.7)	
8.5 Fr	445 (42.2)	180 (21.6)	
10 Fr	98 (9.3)	32 (3.8)	
7 Fr inside stent	11 (1.0)	15 (1.8)	0.16^b^
SEMS	1(0.1)	169 (20.3)	< 0.001^f^
Uncovered	0(0)	154 (18.5)	
Fully covered	1(0.1)	14 (1.7)	
Partial covered	0 (0)	1(0.1)	
**Diameter of SEMS**^**i**^			< 0.001^f^
10 mm	0 (0)	120 (14.4)	
8 mm	1 (0.1)	54 (6.5)	
6 mm	0 (0)	4 (0.5)	
Total no. of stenting per ERCP, Mean (range)	1.0 (1–2)	1.2 (1–4)	< 0.001^a^
Detail of benign and malignant group	See TABLE 4	See TABLE 5	
Size of bile duct stone, mean (SD), mm	9.3 (4.9)		
Number of bile duct stones, Mean (SD)	2.1 (1.4)		
Multiple bile duct stones (≥ 10)	42 (5.9)		
**Stage in malignant group**^**j**^			
Stage 0		4 (0.5)	
Stage I (I + IA + IB)		110 (13.2)	
Stage II (II + IIA + IIB)		138 (16.7)	
Stage III (III + IIIA + IIIB + IIIC)		212 (25.5)	
Stage IV (IV + IVA + IVB + IVC)		394 (43.7)	
Not known		5 (0.6)	
Chemotherapy after stent placement		190 (23.2)	
Time to surgery^k^, Mean ± SD, d		29.0 ± 23.8	
**Gold standard for final diagnosis**			< 0.001^f^
Clinical follow up^l^	977 (92.7)	312 (37.5)	
Surgery	62 (5.9)	187 (22.5)	
Autopsy	4(0.4)	2 (0.2)	
Pathology of ERCP^m^	5 (0.5)	146 (17.5)	
Pathology of EUS-FNA	1 (0.1)	101 (12.1)	
Biopsy	5 (0.5)	0 (0)	
Biopsy from metastasis	0 (0)	50 (6.0)	
Patients who underwent surgery after EBD	100 (9.5)	67 (8.0)	0.27^b^

Unless indicated otherwise, data are presented as n (%). Of note, percentages may not add up to 100% because of rounding.

*SD* standard deviation, *EST* endoscopic sphincterotomy, *SEMS* self-expandable metallic stent, *ERCP* endoscopic retrograde cholangiopancreatography, *EUS-FNA* endoscopic ultrasound-guided fine needle aspiration.

^a^Mann–Whitney test.

^b^Chi-square test.

^c^Tokyo Guidelines 2018: diagnostic criteria and severity grading of acute cholangitis.

^d^PS placement after balloon sphincteroplasty only, large incision of EST and multiple placement of PS without EST (small incision) were unintentionally not included in this series.

^e^Hepaticojejunal anastomosis (n = 15), Post-transpapillary placement by self-expandable metallic stent (n = 34), Post intraductal placement by self-expandable metallic stent (n = 4).

^f^Fisher’s exact test.

^g^Only distal stricture was included and non-stricture was excluded.

^h^Gastrojejunostomy (n = 4), Reconstruction of the esophagus (n = 2), Duodenoplasty (n = 1), Choledocho-duodenostomy (n = 1).

^i^Multiple placement is included.

^j^Based on the Union for International Cancer Control on TNM Classification of Malignant Tumors—8th edition.

^k^Defined as the duration from the initial day of ERCP to the day of surgery. Patients with neoadjuvant chemotherapy were excluded.

^l^Clinical follow up for at least 6 months when surgical resection was not indicated or other pathological method could not be performed because of a benign diagnosis or inoperable malignant disease.

^m^Brush cytology and/or intraductal biopsies, in some cases using a spyglass, were performed during ERCP.

### Comparison of the outcomes between the benign and malignant groups

**Figure 2:**
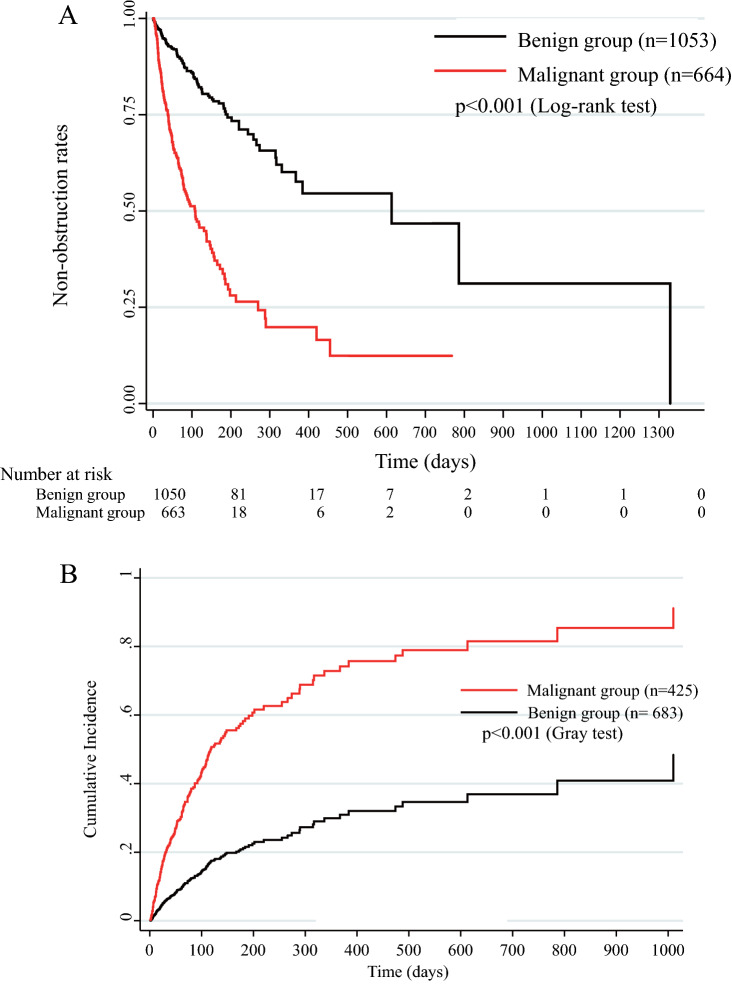
Comparison of the benign and malignant groups in terms of nonobstruction and recurrent biliary obstruction (RBO) rates using Univariate Kaplan–Meier analysis (**A**) and Multivariate Competing-risks model (**B**). (**A**) In the Kaplan–Meier analysis for nonobstruction rates, patients who died were censored, and those in whom self-expandable metallic stents (SEMS) were placed were excluded. (**B**) In the competing risk model for RBO rates, mortality was assigned as the competing risk, and the other independent factors (Table 3) were assigned as covariates. Cases in which SEMSs were placed were included.

The malignant group had significantly lower median PRBO with 95% CI [108 (79–138) days vs. 613 (367–not applicable) days, P < 0.001; Fig. 2A and Table 2]; significantly lower technical success rate of stent insertion (91.7% vs. 96.2%, P < 0.001; Table 2); and significantly lower functional success rate of PS insertion (80.2% vs. 94.5%, P < 0.001; Table 2) the benign group, even in cases of altered gastrointestinal anatomy (61.4% vs. 80.0%, P < 0.001; Table 2). The time to 50% decrease or normalization of the bilirubin level with PS placement was significantly longer in the malignant group than in the benign group [4.4 (3.4) days vs. 3.3 (2.2) days, P = 0.001; Table 2]. The malignant group had lower nonobstruction rates than the benign group after PS placement at 3 months (65.8% vs. 88.7%, P = 0.008); 6 months (44.5% vs. 79.1%, P = 0.005); and 12 months (25.7% vs. 63.0%, P < 0.001) (Table 2). With regard to the adverse events after PS placement, compared with the benign group, the malignant group had higher rates for early (< 30 days) causes of RBO (12.4% vs. 3.9%, P < 0.001); late (≥ 31 days) causes of RBO (18.1% vs. 7.6%, P < 0.001); sludge formation (17.8% vs. 7.9%, P < 0.001); proximal symptomatic migration (0.6% vs. 0%, P = 0.017); and hemobilia (1.7% vs. 0.1%, P < 0.001) (Table 2). In addition, some cases in the benign group that underwent PS placement showed luminal obstruction by sludge without acute cholangitis (Supplementary Fig. S1 online). In contrast, the rate of distal symptomatic migration was higher in the benign group than in the malignant group (3.4% vs. 1.7%, P = 0.02) (Table 2). In the benign group, distal symptomatic migration was significantly higher in 7-Fr PS than in 8.5-Fr and 10-Fr PS (P = 0.001; Supplementary Table S1 online), and there was no significant change in distal symptomatic migration in the malignant group (P = 0.110; Supplementary Table S1 online). No statistically significant differences were noted between the benign and malignant groups with respect to adverse events, other than RBO, and there were no cases of nonobstructive cholangitis and bleeding after stent insertion (Table 2).

**Table 2:** Outcomes and adverse events between benign and malignant groups.

	Benign group (n = 1,054)	Malignant group (n = 833)	*p* value
**Technical success rates**^**a**^**, % (95% CI)**	96.2 (95.0–97.2)	91.7 (89.8–93.4)	< 0.001^b^
Normal anatomy	97.9 (96.9–98.7)	94.0 (92.2–95.4)	< 0.001^b^
Altered gastrointestinal anatomy	80.0 (71.3–87.0)	61.4 (49.0–72.8)	0.006^b^
**Functional success rates**^**c**^	94.5 (92.9–95.8)	80.2 (77.4–82.9)	< 0.001^b^
PS	94.5 (92.9–95.8)	78.7 (75.3–81.8)	< 0.001^b^
7 Fr inside stent	100 (71.5–100)	78.6 (49.2–95.3)	0.23^d^
SEMS	1/1 (100)	86.4 (80.3–91.2)	1.00^d^
**Time to functional success**^**c**^**, Mean ± SD, days**			
PS	3.3 ± 2.2	4.4 ± 3.4	0.001^e^
7 Fr inside stent	7.5 ± 5.0	5.1 ± 4.8	0.38^e^
SEMS		4.7 ± 3.5	
**Median time to RBO**^**f**^**, (IQR), days**			
PS	613 (191–1,329)	108 (39–270)	< 0.001^g^
7 Fr inside stent	112 (92–122)	20 (13–42)	0.23^g^
SEMS		220 (94–488)	
**Nonobstruction rates, % (95% CI)**			
3 months by PS	88.7 (86.2–90.8)	65.8 (61.2–69.9)	0.008^b^
6 months by PS	79.1 (74.4–83.0)	44.5 (36.6–52.0)	0.005^b^
12 months by PS	63.0 (53.2–71.3)	25.7 (15.4–37.3)	< 0.001^b^
3 months by 7 Fr inside stent	90.0 (47.3–98.5)	43.3 (7.5–76.3)	0.015 ^b^
6 months by 7 Fr inside stent	N.A	N.A	
12 months by 7 Fr inside stent	N.A	N.A	
3 months by SEMS		77.5 (69.6–83.6)	
6 months by SEMS		52.5 (42.2–61.7)	
12 months by SEMS		36.1 (25.0–47.3)	
**The timing of RBO**			
Early (within 30 days)	41 (3.9)	105 (12.4)	< 0.001^b^
Late (≥ 31 days)	80 (7.6)	151 (18.1)	< 0.001^b^
**Cause of RBO**			
Sludge	83 (7.9)	151 (18.1)	< 0.001^b^
PS	80 (7.6)	143 (17.2)	< 0.001
7Fr inside stent	3 (0.3)	5 (0.6)	0.31^d^
SEMS	0(0)	3 (0.4)	0.09^d^
Tumor ingrowth with SEMS		45 (5.4)	
Tumor overgrowth with SEMS		20 (2.4)	
Symptomatic migration in distal	36 (3.5)	16 (2.0)	0.06^b^
PS	36 (3.4)	14 (1.7)	0.020^b^
7Fr inside stent	0 (0.0)	0 (0.0)	
SEMS		2 (0.2)	
Symptomatic migration in proximal with PS	0 (0)	5 (0.6)	0.017^d^
Hemobilia	1 (0.1)	14 (1.7)	< 0.001^d^
PS	1 (0.1)	10 (1.2)	0.003^d^
7Fr inside stent	0 (0.0)	0 (0.0)	
SEMS		4 (0.5)	
Food impaction	1 (0.1)	5 (0.6)	0.09^d^
PS	1 (0.1)	0 (0)	
7Fr inside stent	0 (0.0)	0 (0.0)	
SEMS	0 (0.1)	5 (0.6)	
Kinking of bile duct with SEMS		1 (0.1)	
Other	0 (0)	2 (0.2)	
**Adverse events other than RBO**^**h**^			
Pancreatitis	36 (3.4)	28 (3.4)	0.95^b^
Mild/ severe	32 (3.0) / 4(0.4)	21 (2.5) / 7 (0.8)	
Cholecystitis	0 (0)	1 (0.1)	0.44^d^
Severe		1 (0.1)	
Non-occlusion cholangitis	32 (3.0)	125 (15.0)	< 0.001^b^
Moderate	32 (3.0)	125 (15.0)	
Bleeding	12 (1.13)	1 (0.1)	0.009^d^
Mild	12 (1.13)	1 (0.1)	
Ulceration		1 (0.1)	0.44^d^
Moderate		1 (0.1)	
Penetration	1 (0.1)	1 (0.1)	1.00^d^
Mild	1 (0.1)	1 (0.1)	
Perforation	1 (0.1)	4 (0.5)	0.18^d^
Mild/severe	1 (0.1) / 0	3 (0.4) / 1 (0.1)	
**Adverse events associated with stenting**^**h**^			
Bleeding with scope	4 (0.4)	4 (0.5)	0.74^d^
Mild/severe	4 (0.4) / 0	3 (0.4) / 1 (0.1)	
Desaturation of oxygen	1 (0.1)	0 (0)	1.00^d^
Mild	1 (0.1)		

Unless indicated otherwise, data are presented as n (%). Of note, percentages may not add up to 100% because of rounding.

*PS* plastic stent, *SEMS* self-expandable metallic stent, *RBO* recurrent biliary obstruction, *IQR* interquartile range, *CI* confidence interval, *NA* not applicable.

^a^All cases which were requested for endoscopic biliary drainage, including endoscopic nasobiliary drainage.

^b^Chi-square test.

^c^50% decrease in or normalization of the bilirubin level, if biliary stenting was successful.

^d^Fisher’s exact test.

^e^Mann–Whitney test.

^f^Estimated by Kaplan–Meier method.

^g^Log-rank test.

^h^Including self-expandable metallic stent.

### Risk factors for RBO using multivariate competing risk regression analysis

In the multivariate competing risk regression analysis, a significantly longer PRBO was observed with SEMS placement [SHR = 0.37, P = 0.001]; use of 8.5-Fr PS (SHR = 0.58, P = 0.030); and Billroth II anatomy (SHR = 0.56 × 10^−5^, P < 0.001). In contrast, a significantly shorter PRBO was observed in the malignant group (SHR = 3.58, P < 0.001; Table 3 and Fig. 2B).

**Table 3:** Multivariate analysis of recurrent biliary obstruction in benign and malignant groups using competing-risks regression.

RBO = 225, Competing^a^ = 56, Censored = 827	Multivariate competing-risks regression^b^ (n = 1,108)		
Independent variable	SHR	95% CI	*p* value^c^
Age	1.00	0.99–1.02	0.59
Men	1.01	0.74–1.38	0.96
BMI	1.01	0.98–1.04	0.69
Antithrombotic agents	1.27	0.88–1.82	0.20
Serum total bilirubin	1.02	1.00–1.05	0.09
Grade of acute cholangitis	1.06	0.90–1.24	0.49
Periampullary diverticulum	0.80	0.55–1.17	0.24
Intradiverticular papilla	0.72	0.28–1.86	0.50
SEMS	0.37	0.21–0.66	0.001
7Fr inside stent	1.72	0.45–6.48	0.43
10Fr-Plastic stent	Reference	–	–
8.5Fr-Plastic stent	0.58	0.35–0.95	0.030
7Fr-Plastic stent	0.86	0.50–1.49	0.59
Total number of stenting	1.88	0.96–3.68	0.07
**Location of biliary stricture**			
Other^d^	Reference	–	–
Distal	1.26	0.66–2.38	0.48
Perihilar	1.51	0.65–3.51	0.34
Non-stricture	1	(Omitted because of collinearity)	
**Length of stricture**			
< 10 mm	Reference	–	–
10–20 mm	0.83	0.45–1.54	0.56
20–30 mm	0.63	0.34–1.19	0.16
30–40 mm	0.84	0.41–1.76	0.65
> 40 mm	0.68	0.33–1.42	0.31
**Type of major papilla**			
Other type	Reference	–	–
Naïve papilla	1.82	0.27–12.22	0.54
Post EST	2.25	0.31–16.29	0.42
Post plastic stenting	2.08	0.31–13.76	0.45
**Altered gastrointestinal anatomy**			
Other^e^	Reference	–	–
Normal	2.43	0.34–17.33	0.37
Billroth I	6.37	0.87–46.74	0.07
Billroth II	0.56 × 10^–5^	0.06 × 10^–5^–4.94 × 10^–5^	< 0.001
Pancreaticoduodenectomy	0.94	0.03–29.14	0.97
Roux-en-Y with gastrectomy	2.86	0.36–22.47	0.32
Malignant Group	3.58	2.35–5.43	< 0.001

*SHR* subdistribution hazard ratio, *RBO* recurrent biliary obstruction, *BMI* body mass index, *SEMS* self-expandable metallic stent.

^a^Competing event was defined as patient’s death after stent placement.

^b^Fine and Gray model.

^c^Gray test.

^d^Intrahepatic stricture (n = 3), Diffuse stricture (n = 7).

^e^Gastrojejunostomy (n = 4), Roux-en-Y with hepaticojejunostomy (n = 2), Reconstruction of the esophagus (n = 2), Duodenoplasty (n = 1), Choledocho-duodenostomy (n = 1).

### Risk factors for RBO and the best period to replace a PS in benign diseases

**Figure 3:**
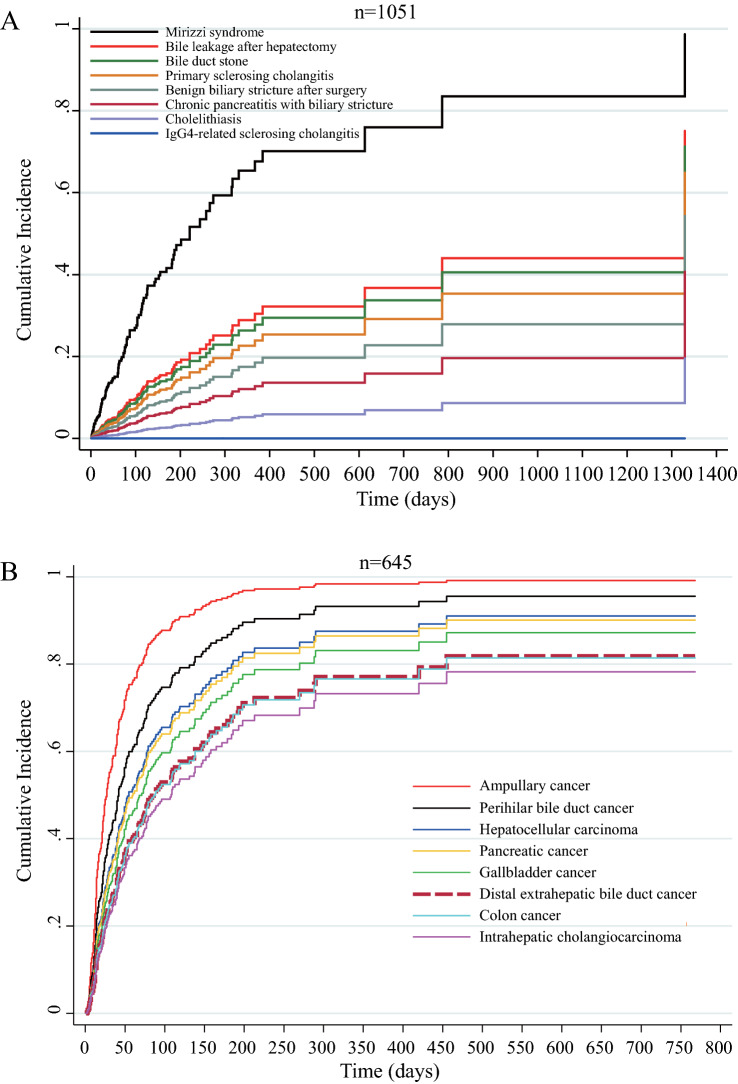
Competing risk model for recurrent biliary obstruction rates for each patient with (**A**) benign and (**B**) malignant diseases were compared. Mortality was assigned as the competing risk, and the other benign and malignant diseases (Tables 4 and 5) were assigned as covariates. Cases in which self-expandable metallic stents were placed were excluded.

In the benign group, a long PRBO was observed with the use of 8.5-Fr PS (SHR = 0.58, P = 0.006; Table 4). Compared with the results shown in Table 3, Billroth II anatomy did not affect PRBO. Among the benign diseases, a significantly long PRBO was seen in IgG4-related sclerosing cholangitis (SHR = 0.07 × 10^−8^, P < 0.001; Table 4 and Fig. 3A), whereas a short PRBO was seen in Mirizzi syndrome (SHR = 3.32, P = 0.030; Table 4 and Fig. 3A). The longest PRBO after the first quartile of a non-RBO period was 1,329 days in chronic pancreatitis with biliary stricture (Table 4). In cases with bile duct stone, the median PRBO was 613 days (Table 4). Mirizzi syndrome cases had the shortest PRBO of 25 days after the first quartile of a non-RBO period, with a median PRBO of 63 days (Table 4). Overall, in the benign group, the first quartile and median period of a non-RBO period were 191 and 613 days, respectively (Table 4).

**Table 4:** Recurrent biliary obstruction of plastic stent in benign disease.

RBO = 121, Competing^a^ = 17, Censored = 913	Multivariate competing-risks regression^b^ (n = 1,051)			The best period to replace plastic stent^c^ (n = 1,053)	
Raw number between benign group (n = 1,054)	SHR	95% CI	*p* value	First quartile^d^ of non-RBO period (95% CI), days	Median time to RBO (95% CI), days
8.5-Fr Plastic stent	0.58	0.39–0.85	0.006	–	–
Billroth II	7.48	0.93–59.94	0.06	–	–
Bile duct stone, 714 (67.7)	0.63	0.23–1.75	0.38	266 (152–317)	613 (315)
Benign biliary stricture after surgery^e^, 87(8.3)	0.52	0.18–1.54	0.24	185 (145–331)	NA (244)
Cholelithiasis, 67 (6.4)	0.12	0.01–1.13	0.06	NA	NA
Chronic pancreatitis with biliary stricture, 49 (4.7)	0.32	0.10–1.08	0.07	1,329 (118)	NA
Mirizzi syndrome, 47 (4.5)	3.32	1.12–9.85	0.030	25 (20–49)	63 (33–221)
Bile leakage after hepatectomy, 23 (2.2)	1.18	0.26–5.38	0.83	98 (5)	98 (98)
Primary sclerosing cholangitis, 20 (1.9)	0.67	0.14–3.23	0.62	80 (46)	NA (48)
IgG4-related sclerosing cholangitis, 17 (1.6)	0.08 × 10^–8^	0.02 × 10^–8^–0.24 × 10^–8^	< 0.001	NA	NA
Other benign disease^f^, 30 (2.9)	Reference	–	–	–	–
Overall benign	–	–	–	191 (145–258)	613 (367–NA)

Unless indicated otherwise, data are presented as n (%). Of note, percentages may not add up to 100% because of rounding. After the initial multivariate analysis (Table 3), the significant variables were adopted in the next multivariate analyses for benign disease.

*SHR* subdistribution hazard ratio, *RBO* recurrent biliary obstruction, *CI* confidence interval, *NA* not applicable.

^a^Competing event was defined as patient’s death after stent placement.

^b^Fine and Gray model.

^c^Estimated by Kaplan–Meier method.

^d^Estimated by interquartile range.

^e^Hepato-biliary-pancreatic surgery.

^f^Hepatic cyst (n = 5), liver cirrhosis (n = 4), bile leakage after liver transplantation (n = 4), confluence stone (n = 4), benign biliary dilation of bile duct (n = 3), anomalous arrangement of the pancreaticobiliary duct (n = 2), normal bile duct (n = 2), adenomyomatosis of the papilla of Vater (n = 1), bile leakage after cholecystectomy (n = 1), chronic inflammation of the papilla of Vater (n = 1), hemobilia with benign disease (n = 1), hepatic abscess (n = 1), urinary tract infection (n = 1).

### Risk factors for RBO and the best period to replace a PS in malignant diseases

In the malignant group, a long PRBO was observed in cases that received chemotherapy after stent placement (SHR = 0.69, P = 0.016; Table 5). Conversely, unlike the results shown in Tables 3 and 4, the use of 8.5-Fr PS did not affect PRBO (Table 5). In addition, cancer stage was not associated with PRBO (Table 5). Among the malignant diseases, a significantly short PRBO was seen in cases of pancreatic cancer (SHR = 2.06, P = 0.041); perihilar bile duct cancer (SHR = 2.69, P = 0.006); and ampullary cancer (SHR = 3.78, P = 0.001) (Table 5 and Fig. 3B). The longest PRBO after the first quartile of a non-RBO was 73 days in gallbladder cancer (Table 5). In cases with colon cancer, the median PRBO was 420 days (Table 5). Cases of ampullary cancer had the shortest PRBO of 22 days after the first quartile of a non-RBO, with a median PRBO of 40 days (Table 5). Overall, in the malignant group, the first quartile and median period of a non-RBO were 39 and 108 days, respectively (Table 5).

**Table 5:** Recurrent biliary obstruction of plastic stent in malignant disease.

RBO = 187, Competing^a^ = 57, Censored = 401	Multivariate competing-risks regression^b^ (n = 645)			The best period to replace plastic stent or change over to SEMS^c^ (n = 664)	
Raw number between malignant group with plastic stent (n = 664)	SHR	95% CI	*p* value	First quartile^d^ of non-RBO period (95% CI), days	Median time to RBO (95% CI), days
8.5-Fr Plastic stent	0.98	0.70–1.39	0.92	–	–
Billroth II	0.91	0.23–3.58	0.89	–	–
**Stage**^e^					
0/I/II	Reference	–	–	–	–
III/IV	0.95	0.69–1.31	0.77	–	–
Chemotherapy after stent placement	0.69	0.50–0.93	0.016	–	–
Pancreatic cancer, 265 (31.8)	2.06	1.03–4.12	0.041	32 (22–42)	92 (55–172)
Perihilar bile duct cancer, 147 (17.7)	2.69	1.32–5.46	0.006	27 (20–49)	77 (49–131)
Distal extrahepatic bile duct cancer, 102 (12.2)	1.28	0.55–3.01	0.57	45 (21–89)	N.A (65)
Gallbladder cancer, 62 (7.4)	1.55	0.65–3.67	0.32	73 (14–118)	118 (73)
Hepatocellular carcinoma, 54 (6.5)	1.84	0.85–3.98	0.12	55 (17–82)	94 (65–137)
Colon cancer, 53 (6.4)	1.24	0.51–3.04	0.63	48 (25)	420 (52)
Ampullary cancer, 52 (6.2)	3.78	1.70–8.44	0.001	22 (7–27)	40 (23–109)
Intrahepatic cholangiocarcinoma, 30 (3.6)	1.12	0.40–3.07	0.83	107 (14–156)	156 (52)
Other malignant disease^f^, 63 (7.6)	Reference	–	–	–	–
Overall malignancy	–	–	–	39 (29–46)	108 (79–138)

Unless indicated otherwise, data are presented as n (%). Of note, percentages may not add up to 100% because of rounding. After the initial multivariate analysis (Table 3), the significant variables were adopted in the next multivariate analyses for malignant disease.

*SEMS* self-expandable metallic stent, *SHR* subdistribution hazard ratio, *RBO* recurrent biliary obstruction, *CI* confidence interval.

^a^Competing event was defined as patient’s death after stent placement.

^b^Fine and Gray model.

^c^Estimated by Kaplan–Meier method.

^d^Estimated by interquartile range.

^e^Based on the Union for International Cancer Control’s TNM Classification of Malignant Tumors—8th edition.

^f^Breast cancer (n = 12), gastric cancer (n = 11), esophageal cancer (n = 9), lymphoma (n = 8), ovarian cancer (n = 5), intraductal papillary neoplasm of the bile duct (n = 4), intraductal papillary mucinous carcinoma (n = 3), parotid gland cancer (n = 3), thyroid cancer (n = 3), bladder cancer (n = 2), duodenal cancer (n = 2), neuroendocrine tumor of papilla (n = 2), renal cell carcinoma (n = 2), Barrett's cancer (n = 1), laryngeal cancer (n = 1).

## Discussion

In the present study, we evaluated PRBO after PS or SEMS placement and evaluated the various factors related to RBO, according to each disease. Malignant diseases significantly differed from benign diseases, particularly with respect to PRBO, nonobstruction rate, cause of RBO, and technical and functional success rates. The multivariate competing risk analysis revealed that the use of SEMS, 8.5-Fr PS, and Billroth II anatomy was the factor that significantly prolonged PRBO. In contrast, the factor that significantly shortened PRBO was the malignant disease. Among the benign diseases, the factor that significantly prolonged PRBO was IgG4-related sclerosing cholangitis and 8.5-Fr PS, not Billroth II anatomy. In contrast, Mirizzi syndrome was the only significant factor that shortened PRBO. Among the malignant diseases, the factors that significantly shortened PRBO were pancreatic cancer, perihilar bile duct cancer, and ampullary cancer, not the use of 8.5-Fr PS and Billroth II anatomy. Chemotherapy after PS insertion was the only factor that prolonged PRBO.

Although SEMS has shown the advantage of a longer patency than PS^5,9,13,17^, its use upon initial insertion had been hesitated for the following cases: (1) placement of an uncovered SEMS before pathologic diagnosis mentioned differentiation between benign and malignant conditions^5,18^, (2) perihilar bile duct cancer before a decision to choose between surgery or nonsurgery, based on the clinical stage^5,16^, (3) operable malignant perihilar stricture in specific situations, such as acute cholangitis, ≤ 30% predicted volume of liver remnant following surgery^5^, (4) intake of anticoagulant agents and placement of fully covered SEMS without EST^5,15,29,30^, (5) more cost-effective use of PS in patients with short life expectancy^1,16,17^, (6) dilemma between unilateral and bilateral drainage for palliative EBD in perihilar bile duct cancer, such as Bismuth II–IV type^5^, and (7) benign diseases^1,5,16^.

In the present study, benign diseases without Mirizzi syndrome showed a long PRBO, suggesting that the interval to replace a PS may be feasible at 191 days after the first quartile, with a median of 613 days (Table 4), for patients with special circumstances, such as those with severe complications and who could not undergo EST and clearance of bile duct stone or cholecystectomy and those with benign biliary stricture from IgG4-related or chronic pancreatitis. The reason for a longer PRBO in benign nonbiliary stricture from bile duct stone or other benign biliary strictures than in malignant diseases might be the tighter stricture in malignant cases; even in benign diseases that develop luminal obstruction with sludge, the bile flow along with PS may prevent RBO (Supplementary Fig. S1 online). In fact, our results showed that the number of cases with sludge as the cause of RBO was significantly fewer in benign than in malignant diseases (Table 1). Moreover, we believed that prolonged factor for PRBO was not only the presence or absence of the biliary stricture but also the presence of strong stricture such as malignant disease (Supplementary Fig. S1 online) because our multivariate competing risk analysis (Table 4 and Fig. 3A) revealed that “IgG4-related sclerosing cholangitis” coupled with biliary stricture showed significantly prolonged PRBO compared with other benign diseases, including “bile duct stone” coupled with non-stricture. In addition, the multivariate competing risk analysis revealed that the use of 8.5-Fr PS was the factor that significantly prolonged PRBO in the benign group. This result may indicate that 8.5-Fr PS is the best thickness for longer intervals in benign disease because 10-Fr PS may be too thick for the bile flow, along with PS against benign stricture, and 7-Fr PS may involve a risk of distal symptomatic migration (Table 2, Supplementary Table S1 online). Thus, contrary to the published standards within 3–6 months intervals^5,31^, our results indicate that longer intervals for routine exchange of 8.5-Fr PS may be acceptable^32^.

Competing risk analysis showed a shorter PRBO after PS placement for malignant diseases than for benign diseases. For pancreatic cancer, routine PS replacement within 32 days after the first quartile or early change to SEMS may be needed to keep up with the scheduled surgery or chemotherapy. For perihilar bile duct cancer and ampullary cancer, the required interval for PS replacement or changing to SEMS would be within 27 days and 22 days, respectively, after the first quartile.

More recently, SEMS has been recommended to patients who have > 3 months of life expectancy, from the point of view of cost effectiveness and PRBO^1,13,16,19^. However, our study indicated that even patients with pancreatic cancer, perihilar bile duct cancer, and ampullary cancer who have more than 1 month of life expectancy may be recommended to receive SEMS placement. PS had been used during the initial EBD for suspected inoperable malignant biliary strictures in our hospital, because a pathologic diagnosis would be required before chemotherapy, including neoadjuvant chemotherapy^33^^,^ and to repeat ERCP, in case the biopsy material was inadequate for pathologic diagnosis. Initial placement of a PS may be useful in such situations, because initial placement of an uncovered SEMS would render repeated ERCP for pathologic diagnosis difficult^5^. In contrast, it is also easy to replace a fully covered SEMS and PS; however, a fully covered SEMS is more expensive than a PS^34^.

Nowadays, preoperative EBD in distal malignant stricture has not been recommended due to the risk for postoperative pancreatic fistula, except for ongoing cholangitis or severe obstructive jaundice (serum bilirubin ≥ 300 μmol/L)^1,5,10,16,35–37^. However, many institutions in Japan tend to perform preoperative EBD owing to the long wait to surgery (range, 28.0–33.5 days), which, in itself, is a high risk factor for preoperative cholangitis arising out of non-symptomatic obstructive jaundice because preoperative cholangitis is related to postoperative pancreatic fistula^36–39^. A previous randomized controlled trial showed that the mean waiting time to surgery was only 8.4 days or 1.2 weeks^35^. The reason for this prolonged waiting time to surgery in Japan may be the detailed preoperative examinations, including examination of the whole body for complication and the decision on clinical stage and preoperative pathology^38,39^. The present study showed that the frequency of acute cholangitis at initial EBD for malignant disease was 54.7%, with a waiting time to surgery of 29 days. Taking together these results, preoperative EBD using a PS may be proposed and recommended for cases without pancreatic cancer and ampullary cancer, considering the cost effectiveness, because earlier routine replacement of a PS might be recommended for these two diseases^1,13^. In summary, only patients with ongoing cholangitis or severe obstructive jaundice may be required to undergo preoperative EBD with rapid triage to surgery within 22 days for ampullary cancer, including 32 days of pancreatic cancer, after PS placement or may require a fully covered SEMS^1,10^.

Our study had several limitations. First limitation was the retrospective and single-center design of the study. Second, several selection biases may have been included; for example, the choice of the diameter and type of PS were not fixed under a given condition and were left to the preference of the endoscopist. Thus, further prospective study is needed for prolonged PRBO of 8.5-Fr PS in the benign group. Finally, this study contained many confounding background factors that differed among the diseases.

In conclusion, our study supports that stent replacement for the benign group is feasible after 6 months, and the best period to replace or change a PS with a SEMS should be decided on the basis of underlying disease to prevent RBO.

## Supplementary information

Supplementary file 1
