# Supplementary material for: Best period to replace or change plastic stents with self-expandable metallic stents using multivariate competing risk regression analysis
Source: Sci Rep. 2020 Aug 4;10:13080. doi: 10.1038/s41598-020-70081-3 (PMC7403588; doi:10.1038/s41598-020-70081-3)
Supplement: Supplementary file 1 [file 41598_2020_70081_MOESM1_ESM.pdf]

Best Period to Replace or Change Plastic Stents with Self-Expandable Metallic Stents Using Multivariate Competing Risk Regression Analysis

Masafumi Chiba, MD, PhD[1], Masayuki Kato, MD, PhD[1], Yuji Kinoshita, MD[2], Nana Shimamoto, MD[1], Youichi Tomita, MD[2], Takahiro Abe, MD[1], Yosuke Kawahara, MD[1], Seita Koyama, MD[1], Keisuke Kanazawa, MD, PhD[1], Kazuki Takakura, MD, PhD[2], Shintaro Tsukinaga, MD[1], Masanori Nakano, MD, PhD[2], Yuichi Torisu, MD, PhD[2], Hirobumi Toyoizumi, MD, PhD[1], Keiichi Ikeda, MD, PhD[1], Hiroshi Arakawa, MD, PhD[1], Kazuki Sumiyama, MD, PhD[1]

1. Department of Endoscopy, The Jikei University School of Medicine, Tokyo, Japan
2. Division of Gastroenterology and Hepatology, Department of Internal Medicine, The Jikei University School of Medicine, Tokyo, Japan

Correspondence:

Masafumi Chiba

Department of Endoscopy, The Jikei University School of Medicine

3-25-8, Nishi-Shimbashi, Minato-ku, Tokyo 105-8461, Japan

Tel: +81 3 34331111 ext. 3181; Fax: +81 3 34594524

E-mail: ccl0972009720@gmail.com

Supplementary Table S1. Comparison of symptomatic migration in distal between the plastic stent (PS) diameter in benign and malignant groups

	7-Fr PS	8.5-Fr PS	10-Fr PS	*p* value[A]
Benign group, n (%)	21 (2.0)	12 (1.1)	3 (0.3)	.001
Malignant group, n (%)	7 (1.1)	6 (0.9)	1 (0.2)	.110

[A]Fisher's exact test

A

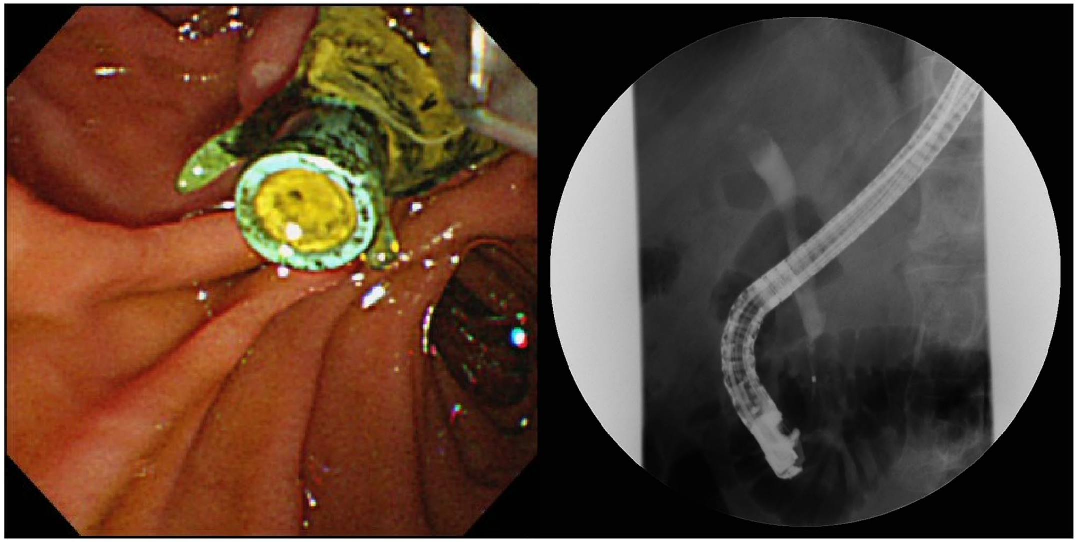

Chronic pancreatitis	
Stricture	Distal, Bi
Exchange interval, day	186
WBC, (/μL)	6500
CRP, (mg/dl)	0.60
T-Bil, (mg/dl)	1.5
AST/ALT, (IU)	34/84
ALP/γ-GTP, (IU)	1776/994

B

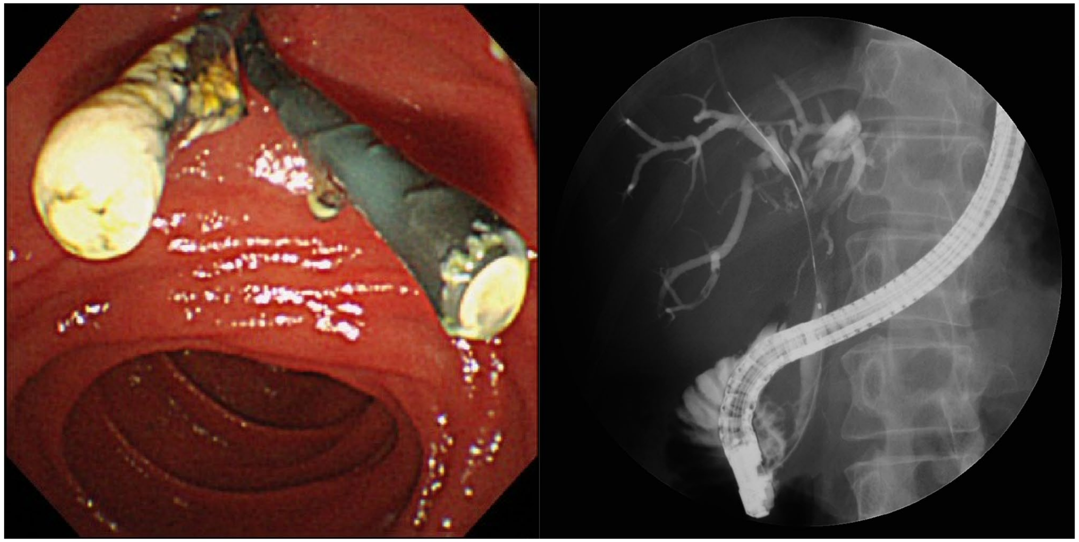

Perihilar bile duct cancer	
Stricture	Perihilar
Exchange interval, day	34
WBC, (/μL)	6200
CRP, (mg/dl)	5.06
T-Bil, (mg/dl)	5.8
AST/ALT, (IU)	159/205
ALP/γ-GTP, (IU)	1545/975

Supplementary Figure S1. Comparison of plastic stent luminal occlusion with sludge between non-RBO benign and RBO malignant cases

A. In a routine exchange case, there is luminal occlusion with sludge under a non-RBO condition (i.e., no jaundice and cholangitis).

B. Luminal occlusion with sludge is seen under RBO (i.e., with jaundice and cholangitis) conditions.

Both serum laboratory test values were taken during the preceding ERCP.

RBO, recurrent biliary obstruction; ERCP, Endoscopic Retrograde Cholangiopancreatography.
